# Optimization protocol for the extraction of 6-gingerol and 6-shogaol from *Zingiber officinale* var. *rubrum* Theilade and improving antioxidant and anticancer activity using response surface methodology

**DOI:** 10.1186/s12906-015-0718-0

**Published:** 2015-07-30

**Authors:** Ali Ghasemzadeh, Hawa Z.E. Jaafar, Asmah Rahmat

**Affiliations:** Department of Crop Science, Faculty of Agriculture, Universiti Putra Malaysia, 43400 Serdang, Selangor Malaysia; Department of Nutrition & Dietetics, Faculty of Medicine & Health Sciences, Universiti Putra Malaysia, 43400 Serdang, Selangor Malaysia

**Keywords:** 6-Gingerol, 6-Shogaol, DPPH activity, *Zingiber officinale* var. *rubrum* Theilade, Response surface methodology

## Abstract

**Background:**

Analysis and extraction of plant matrices are important processes for the development, modernization, and quality control of herbal formulations. Response surface methodology is a collection of statistical and mathematical techniques that are used to optimize the range of variables in various experimental processes to reduce the number of experimental runs, cost , and time, compared to other methods.

**Methods:**

Response surface methodology was applied for optimizing reflux extraction conditions for achieving high 6-gingerol and 6-shogaol contents, and high antioxidant activity in *Zingiber officinale* var. *rubrum Theilade *. The two-factor central composite design was employed to determine the effects of two independent variables, namely extraction temperature (*X*_*1*_:50–80 °C) and time (*X*_*2*_:2–4 h), on the properties of the extracts. The 6-gingerol and 6-shogaol contents were measured using ultra-performance liquid chromatography. The antioxidant activity of the rhizome extracts was determined by means of the 1,1-diphenyl-2-picrylhydrazyl assay. Anticancer activity of optimized extracts against HeLa cancer cell lines was measured using MTT (3-(4,5-dimethylthiazol-2-yl)-2,5-diphenyltetrazolium bromide) assay.

**Results:**

Increasing the extraction temperature and time induced significant response of the variables. The optimum extraction condition for all responses was at 76.9 °C for 3.4 h. Under the optimum condition, the corresponding predicted response values for 6-gingerol, 6-shogaol, and the antioxidant activity were 2.89 mg/g DW, 1.85 mg/g DW, and 84.3 %, respectively. 6-gingerol and 6-shogaol were extracted under optimized condition to check the viability of the models. The values were 2.92 and 1.88 mg/g DW, and 84.0 % for 6-gingerol, 6-shogaol, and the antioxidant activity respectively. The experimental values agreed with those predicted, thus indicating suitability of the models employed and the success of RSM in optimizing the extraction condition. With optimizing of reflux extraction anticancer activity of extracts against HeLa cancer cells enhanced about 16.8 %. The half inhibition concentration (IC_50_) value of optimized and unoptimized extract was found at concentration of 20.9 and 38.4 μg/mL respectively. Optimized extract showed more distinct anticancer activities against HeLa cancer cells in a concentration of 40 μg/mL (P < 0.01) without toxicity to normal cells.

**Conclusions:**

The results indicated that the pharmaceutical quality of ginger could be improved significantly by optimizing of extraction process using response surface methodology.

## Background

Herbs and natural products are precious sources of medicinal compounds and their benefits and importance for healing have been well recognized since ancient times. The characteristics and health effects of natural bioactive compounds, especially from plant sources including spices, have been extensively investigated. Phytochemicals are important compounds found in medicinal plants that are not essential for the normal functioning of the human body, but are active and exert positive effects on health or in amelioration of diseases. Many phytochemicals have been identified though a great many are yet to be identified [1]. According to a report by the World Health Organization, 80 % of the population in developing countries depends on traditional medicine for their primary health care, and 85 % of traditional medicine is derived from plant extracts [2]. In Malaysia, herbs and spices are generally consumed raw and fresh as vegetables (salad), especially by the Malay community. Ginger (*Zingiber officinale* Roscoe) is one of the most widely used spices in the world, especially in Malaysia and locally was known as Halia. Owing to its universal appeal, ginger has spread to most tropical and subtropical countries from China and India, where ginger cultivation has been prevalent, possibly since prehistoric times [3]. In ancient times, ginger was highly valued for its medicinal properties and it played an important role in primary health care in ancient India and China. Ginger contains a variety of pungent and biologically active compounds, primarily 6-gingerol, 6-shogaol, zingerone, phenolics, and flavonoids [4]. Between identified components, 6-gingerol was reported as the most abundant bioactive compound in ginger with various pharmacological effects including antioxidant, analgesic, anti-inflammatory and antipyretic properties [5–8]. The result of recent studies showed that 6-shogaol with lowest concentration in ginger represent more biologically actives compared to 6-gingerol [9–11] Dugasani et al. [12] reported 6-shogaol as a potent anti-inflammatory and antioxidant compounds in ginger. Various methods for the analysis of 6-gingerol and 6-shogaol in ginger extract have been reported [13–15] but, among these, high-performance liquid chromatography (HPLC) is most widely utilized. Extraction prior to component analysis is the main step for the recovery and isolation of bioactive phytochemicals from plant materials. Analysis and extraction of plant matrices are important processes for the development, modernization, and quality control of herbal formulations [13]. In general, the first step of complete extraction is the selection of plant parts and careful preparation of plant extracts, and a thorough review of the existing literature to learn about the most suitable protocols for a specific group of compounds or plant species. Traditionally, the extraction of 6-gingerol and 6-shogaol compounds is accomplished by reflux or Soxhlet extraction [16]. However, prolonged extraction at high temperature may degrade the 6-gingerol and 6-shogaol compounds, and involves high energy cost. Bhattarai et al. [17] recently reported that in acidic media and under high extraction temperature 6-gingerol can be degraded to 6-shogaol. Generally, the widespread use of ginger as a spice, dietary supplement, tea, cream, household remedy, as well as an ingredient of various herbal formulations, requires standardization of ginger formulations. A model for optimizing the most relevant operational parameters is required in order to achieve higher extraction yield. Response surface methodology (RSM) is a collection of statistical and mathematical technique that used to optimize the range of variables in various experimental processes with  reducing the number of experimental runs, cost and time compared to other methods. *Zingiber officinale* var. *rubrum* Theilade is distributed mainly in Peninsular Malaysia, where it is known locally as halia udang, halia merah and halia bara. To the best of our knowledge, no other studies have been undertaken to optimize extraction condition of 6-gingerol and 6-shogaol from *Z.officinale*.var.*rubrum* Theilade. The aim of this study was to optimize the conditions for the extraction of a Malaysian ginger variety *Zingiber officinale *var. *rubrum* Theilade namely Halia bara to achieve high 6-gingerol and 6-shogaol contents and high antioxidant and anticancer capacity by using response surface methodology with a central composite design (CCD).

## Methods

### Plant materials

*Z.officinale *var. *rubrum* Theilade rhizomes were collected from Bentong, Pahang, Malaysia. The samples were identified by herbarium of department of biology, faculty science, University Putra Malaysia. Rhizomes were washed with pure water and were soaked in a Mancozeb solution (0.3 %) for 30 min and were cut into 3–5 cm pieces containing 2 to 3 buds. After cutting, all pieces were planted 6 cm deep into the small pots filled with about 1 kg peat moss. Rhizomes were grown in a glasshouse for two weeks. Afterward, seedlings with 2 or 3 leaves were transplanted into polyethylene bags filled with a soilless mixture composed of burnt rice husk and coco peat (1:1). Ginger is a semi-shade loving plant and needs shade for growth and rhizome production. Then, the plants were grown under glasshouse conditions at the glasshouse complex of Universiti Putra Malaysia (UPM) where daily irradiance was approximately 790 μmol/m^2^/s (light intensity in outside was 1150 μmol/m^2^/s). Relative humidity was 70 ± 5 % and average temperature was 28 ± 1 °C. The plants were harvested after nine month, with the leaves, stems, and rhizomes separated. The rhizomes were shade dried and were powdered using grinder. These powdered materials were used for further analysis.

### Extraction

The optimization procedure for the extraction process focusing on the extraction temperature (*X*_*1*_: 50–80 °C) and extraction time (*X*_*2*_: 2–4 h) was devised based on two factor central composite design, as summarized in Table 1. Rhizomes of *Z.officinale *var. *rubrum* Theilade were harvested and washed with water and shade dried. Ten grams of dried rhizomes were extracted with absolute ethanol (100 mL) for 2–4 h at 50- 80 °C using a reflux apparatus (Table 1). The ginger extracts were filtered through Whatman No.1 filter paper and kept at −20 °C for future analysis.

**Table 1:** Levels of independent variables for reflux extraction condition based on CCD

	Coded				
Variable	−1	0	1	Axial (−α)	Axial (+α)
X_1_: extraction temperature	50	65	80	47.1	82.8
*X*_2_: extraction time	2	3	4	1.8	4.1

### Ultra High Performance Liquid Chromatography (UHPLC) analysis

The UHPLC system (Agilent, Model 1200) with Agilent C_18_ (4.6 × 250 mm, 5 μm) column was used for 6-gingerol and 6-shogaol analysis. In this system two mobile phases including: (A) water and (B) acetonitril (CAN) were used. The column temperature, flow rate and injection volume were adjusted at 48 °C, 1 mL/min, 20 μL. The UV absorbance was measured at 280 nm. To prepare the standard solution 6-gingerol and 6-shogaol (0.0625, 0.125, 0.250, 0.500 and 1 mg/mL) were dissolved in HPLC grade methanol. The linear regression equation were calculated with Y = aX ± b, where X was concentration of 6-gingerol and 6-shogaol and Y was the peak area of 6-gingerol and 6-shogaol obtained from UHPLC. Identification of the compounds was achieved by comparison of retention times with standards, UV spectra and UV absorbance ratios after co-injection of samples and standards. System suitability requirements: Perform at least five replicate injections of 6-gingerol and 6-shogaol. The requirements of the system suitability parameters are : (1) Symmetry factor (A_s_) is not more than 1.5, (2) Percentage of relative standard deviation (RSD) of the retention time (t_r_) for 6-gingerol and 6-shogaol standards is not more than 2.0 %.

### 1,1-Diphenyl-2-picrylhydrazyl (DPPH) assay

The free radical scavenging activity of *Z.officinale* var. *rubrum* Theilade extracts were determined according to the Mensor et al. [18] with some modification. DPPH was dissolved in methanol to give final concentration of 2 mM. Following that, 1 mL of DPPH solution was added to different concentration of curry leaf extracts (20, 40, 60 80 and 100 mg/mL). The mixture was shaked gently and incubated at 28 °C in a dark room for 40 min. For the control, methanol was used as a blank. The absorbance of the samples was read at 517 nm using spectrophotometer. BHT (butylhydroxytoluene) and α-tocopherol, were used as positive controls. The scavenging activity was calculated using the following formula:

1 $$ \%\ \mathrm{inhibition}=\left[\left(\mathrm{absorbance}\ \mathrm{of}\ \mathrm{control}\ \hbox{--}\ \mathrm{absorbance}\ \mathrm{of}\ \mathrm{sample}\right)/\left.\mathrm{absorbance}\ \mathrm{of}\ \mathrm{control}\right)\right]\times 100 $$

### Determination of anticancer activity

Retrieve the frozen cells from liquid nitrogen cell storage tank and thaw the cells in cyrovials rapidly. Carefully transfer the contents of the cyrovial to a centrifuge tube and add 10 ml of pre-warmed media slowly to the cell suspension. Spin down at 1000 rpm for 10 min and gently re-suspend pellet in 10 ml fresh media into culture flask. Incubate in 37 °C humidified incubator supplemented with 5 % CO_2_. After 24 h, the old medium was discard one day after seeding and adds 2-3 ml PBS to cover all surface and discard. Add 1.5-2 ml trypsining solution to cover the flask surface and leaved at room temperature for 3 min until most of the cells detach. Add 10 ml of complete medium. For MTT assay add 100 μl of cell into all the wells various concentrations of optimized (76.9 °C and 3.4 h) and unoptimized (80 °C and 4 h) extracts (10, 20, 40, 80 and 160 μg/mL) and incubate in 37 °C, 5 % CO_2_ incubator for 72 h. Prepare a stock solution of 5 mg/ml MTT (3-(4,5-dimethylthiazol-2-yl)-2,5-diphenyltetrazolium bromide) in Phosphate buffered saline (PBS) and add 20 μL of MTT reagent to cell monolayer. Add 100 μl of DMSO (Dimethyl sulfoxide) to each well and mix thoroughly by pipetting 10–20 times to dissolved the blue formazan crystals. The absorbance of samples was read at 570 nm using ELISA reader.

### Experimental design

RSM software with central composite experimental design (3-level, 2-factorial) was used to investigate and validate extraction parameters affecting the extraction yields of 6-gingerol (*Y*_*1*_), 6-shogaol (*Y*_*2*_) and antioxidant activity (*Y*_*3*_) of *Z.officinale* var. *rubrum* Theilade rhizomes extracts. In this study, 14 experiments were designed and carried out in duplicate with different range of the independent variables, reflux temperature (*X*_*1*_: 50–80 °C) and extraction time (*X*_*2*_: 2–4 h). In order to conduct the experimental design and the statistical analysis the Design Expert software (version 6.0) was used. Analysis of variance (ANOVA) and response surface analysis were used to determine the statistical significance of the model. The adequacy of the model was predicted through the ANOVA (P < 0.05) and regression analysis (R^2^). The relationship between the response and independent variables was demonstrated using response surface plot. Initially, a second-order polynomial model was set up to predict the response variables. The equation is given below:

2 $$ Y={b}_0+{b}_1{X}_1+{b}_2{X}_2+{b_1}^2{X_1}^2+{b_2}^2{X_2}^2+{b}_1{b}_2{X}_1{X}_2 $$

Where *Y* is the predicted dependent variable; *b*_*0*_ is a constant that fixes the response at the central point of the experiment; *b*_*1*_*, b*_*2*_*, b*_*1*_^*2*^*, b*_*2*_^*2*^*,* and *b*_*1*_*b*_*2*_ are the linear, quadratic, and interaction coefficients, respectively. Graphical and numerical optimizations were performed to obtain the optimum conditions and predicted values for the response variables based on the response optimizer.

## Results and Discussion

### Effects of extraction conditions on 6-gingerol and 6-shogaol content

The reflux extraction was designed based on two-factor CCD consisting of extraction temperature (*X*_*1*_: 50–80 °C) and extraction time (*X*_*2*_: 2–4 h) at five levels each (Table 1). The result of response surface methodology demonstrated significant (*P* < 0.05) regression relationships between the independent variables and response variables. A high content of 6-gingerol (2.74 mg/g DW) in the rhizome extract was observed for treatment 3 (Table 2). The current regression analysis also indicated that more than 80 % of the variations could be explained by the models. Analysis of variance for predicted extraction model of 6-gingerol implies that the model is highly significant with a good coefficient of determination (R^2^ = 0.97). The result indicated significant (P < 0.01) quadratic and linear effects of the extraction temperature and time on 6-gingerol content (*Y*_*1*_). However, no interactive effect was observed for the independent variables. The predicted model obtained for *Y*_*1*_ is as follows:

**Table 2:** Experimental data and predicted content obtained for the dependent variables

Treatment	*X*_*1*_	*X*_*2*_	6-gingerol content (mg/g DW)	Predicted content of 6-gingerol	6-shogaol content (mg/g DW)	Predicted content of 6-shogaol	Antioxidant activity %	Predicted content of antioxidant activity %
1 (c)	65	3	1.49	1.52	1.25	1.20	68.0	71.0
2	50	4	1.08	1.21	0.90	1.02	62.5	65.0
3	80	4	2.74	2.80	1.59	1.63	89.0	83.8
4	80	2	1.79	1.73	1.20	1.12	78.0	78.3
5(c)	65	3	1.56	1.52	1.33	1.24	69.0	71.0
6(c)	65	3	1.42	1.52	1.21	1.20	68.0	71.0
7	50	2	1.38	1.33	1.04	1.08	66.0	60.8
8(c)	65	3	1.62	1.52	1.31	1.20	70.0	71.0
9	82.8	3	2.55	2.53	1.32	1.35	84.0	85.6
10	65	4.1	2.00	1.92	1.55	1.48	79.0	75.8
11	47.1	3	1.35	1.28	1.10	1.05	66.8	62.6
12(c)	65	3	1.52	1.52	1.28	1.20	73.0	71.0
13	65	1.8	1.11	1.19	0.82	0.97	66.0	68.0
14(c)	65	3	1.55	1.52	1.24	1.20	71.0	71.0

*c* central point

3 $$ {Y}_1=1.53+0.51{X}_1+0.25{X}_2+0.31{X}_1{X}_2+0.27{X_1}^2+0.009233{X_2}^2 $$

Lack of fit test for the model describes the variation in the data around the fitted model. If the model does not fit the data well, the value of lack of fit will be significant and then proceeding with investigation and optimization of the fitted response surface is likely to give misleading results [19]. In current study, the “Lack of Fit *F* -value” of 4.12 implies that the Lack of Fit is not significant relative to the pure error (Table 3).

**Table 3:** Regression coefficients, R^2^, adjusted R^2^ and *F*-values for dependent variables

	constant	linear	quadratic	Interaction						
Responses	b_0_	b_1_	b_2_	b_1_^2^	b_2_^2^	b_12_	R^2^	R^2^ (adjusted)	Regression (F value)	Lack of fit (F value)
Y_1_	1.53	0.51	0.25	0.27	0.0092	0.31	0.97	0.95	45.91^**^	4.12 ^n.s^
Y_2_	1.2	0.15	0.12	0.021	0.024	0.15	0.93	0.92	15.58^**^	1.39 ^n.s^
Y_3_	71.88	9.22	2.78	3.16	1.11	4.63	0.96	0.94	15.79^**^	1.24 ^n.s^

*n.s* non significant

** = significant at *p* < 0.01

**Fig. 1:**
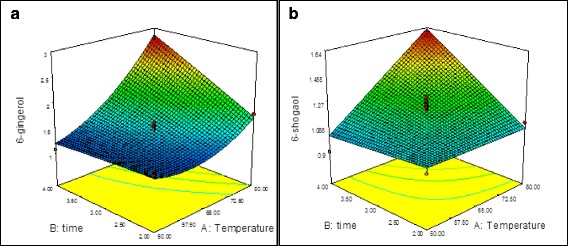
Response surface plot showing the relationship between the 6-gingerol (**a**) and 6-shogaol (**b**) content with the extraction temperature and time

The response surface plot in Fig. 1a shows the relationship between the 6-gingerol content and the extraction temperature, as well as time, illustrating that as the temperature (47.1–82.8 °C) and time (1.8–4 h) increased, the 6-gingerol content increased. Upon increasing the extraction time from 2 to 4 h at 80 °C, the 6-gingerol content increased from 1.79 to 2.74 mg/g DW.

The result of previous study demonstrated that ß-hydroxy keto presence in gingerols structure is a sensitive to high temperature and promotes dehydration of gingerols at high temperature [17]. The present results are consistent with observations by Bak et al. [16] who reported that when ginger was extracted at room temperature, the 6-gingerol content tended to decrease as the drying temperature increased up to 60 °C, whereas drying at 80 °C resulted in an increase in the 6-gingerol yield. The origin of the enhanced 6-gingerol content at 80 °C is not clear at this moment.

Analysis of variance for predicted extraction model of 6-shogaol implies that the model is highly significant with a good coefficient of determination (R^2^ = 0.93) (Table 3). Significant (*P* <0.05) quadratic effects of the extraction temperature and time on the 6-shogaol content (*Y*_*2*_) were observed. The predicted model obtained for *Y*_*2*_ is as follows:

4 $$ {Y}_2=1.20+0.15{X}_1+0.12{X}_2+0.15{X}_1{X}_2 $$

**Fig. 2:**
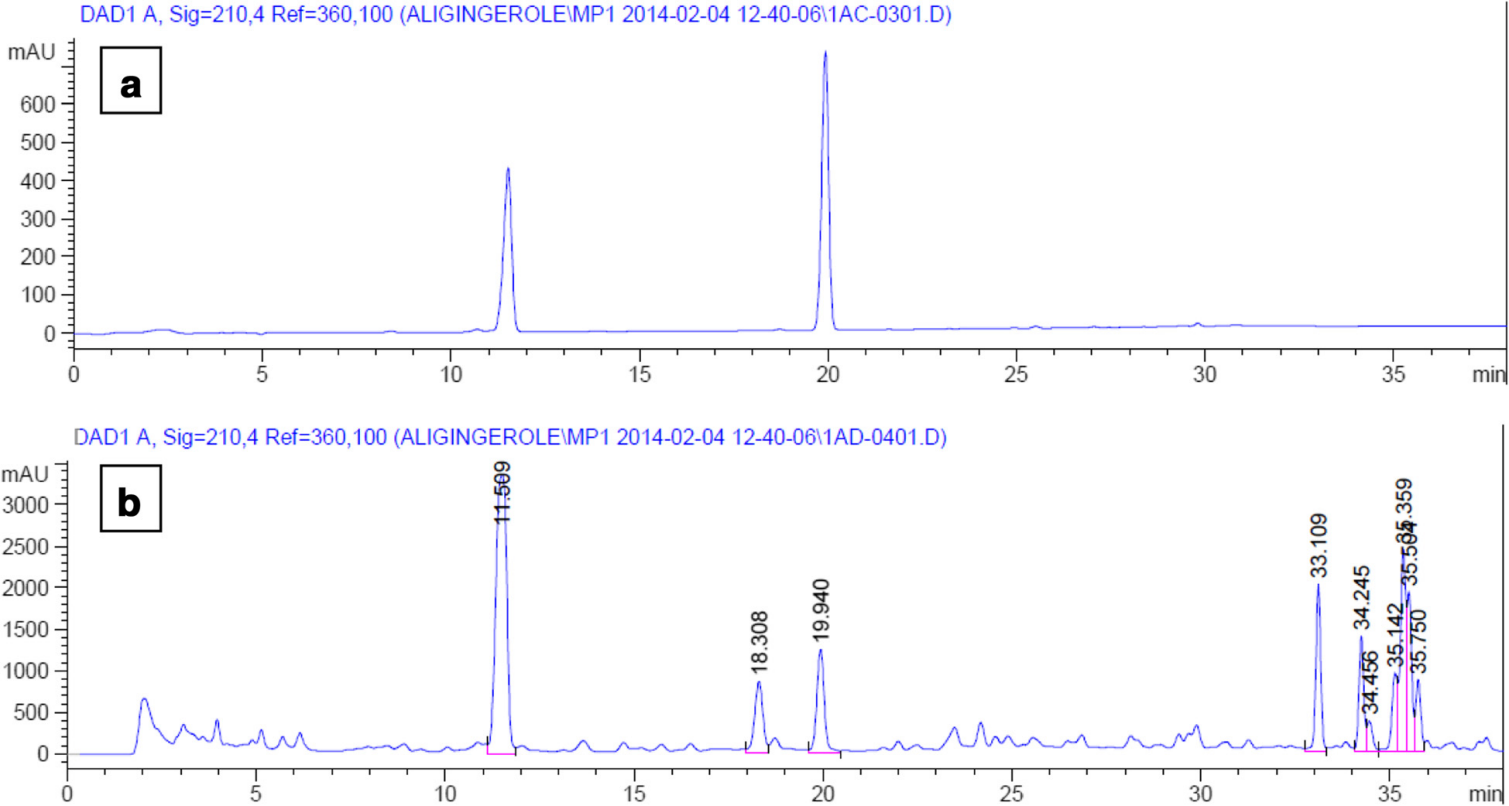
**(a)** UHPLC full chromatogram of 6-gingerol standard solution (0.5 mg/ml) at tr = 11.509 min and 6-shogaol standard solution (0.4 mg/ml) at tr = 19.940 min, (**b**) UHPLC full chromatogram of ethanol extract of *Z.officinale* Roscoe var. rubrum dried rhizome powder showing peak corresponding to 6-gingerol and 6-shogaol standard solution. Extraction condition: at 80 °C for 4 h

The model F-value of 15.58 obtained for the 6-shogaol content implies that the model is significant, with only a 0.07 % probability that such a large “model F-value” can occur owing to noise. The “Lack of Fit *F* -value” of 1.39 implies that the Lack of Fit is not significant for the predicted model (Table 3). In the current study, treatment 3 yielded a high content of 6-shogaol in the ginger extract, with a value of 1.59 mg/g DW. The predicted content of 6-shogaol for this treatment was 1.63 mg/g DW, which was close to the experimental value (Table 2). Figure 1b shows the response surface relationship between the 6-shogaol content and the extraction temperature and time. The obtained results were consistent with previous studies, which reported that the 6-shogaol content increased with higher drying and extraction temperatures and the lowest 6-shogaol content was achieved when the freeze-dried ginger was extracted at a low temperature (30 °C), whereas the highest 6-shogaol content was obtained when the ginger was dried and extracted at a high temperature (80 °C) [17]. The results demonstrate that the amount of 6-shogaol in rhizomes extract depends more strongly on the extraction temperature than the extraction time. Figure 2 shows UHPLC chromatograms of ginger ethanol extract and standards.

### Effects of extraction conditions on antioxidant activity

The DPPH stable free radical method is an easy, rapid, and sensitive method for evaluation of free radical scavenging antioxidants. The results of the DPPH assay showed that the antioxidant activity for all the extracts was more than 62.5 % (Table 2). The DPPH activity was particularly high for the extracts subjected to treatment 3 (80 °C, 4 h), with a value of 89 %. On the other hand, the regression equation obtained using the antioxidant activity (*Y*_*3*_) as the response variable was also significantly (*P* < 0.05) related to the variation of the independent variables. The coefficient of determination (R^2^) obtained was 0.96 (Table 3). Significant (*P* < 0.05) linear and quadratic effects of the extraction temperature and time on the antioxidant capacity were observed. The predicted model obtained for *Y*_*3*_ is given below:

5 $$ {Y}_3=71.88+9.22{X}_1+2.78{X}_2+4.63{X}_1{X}_2 $$

**Fig. 3:**
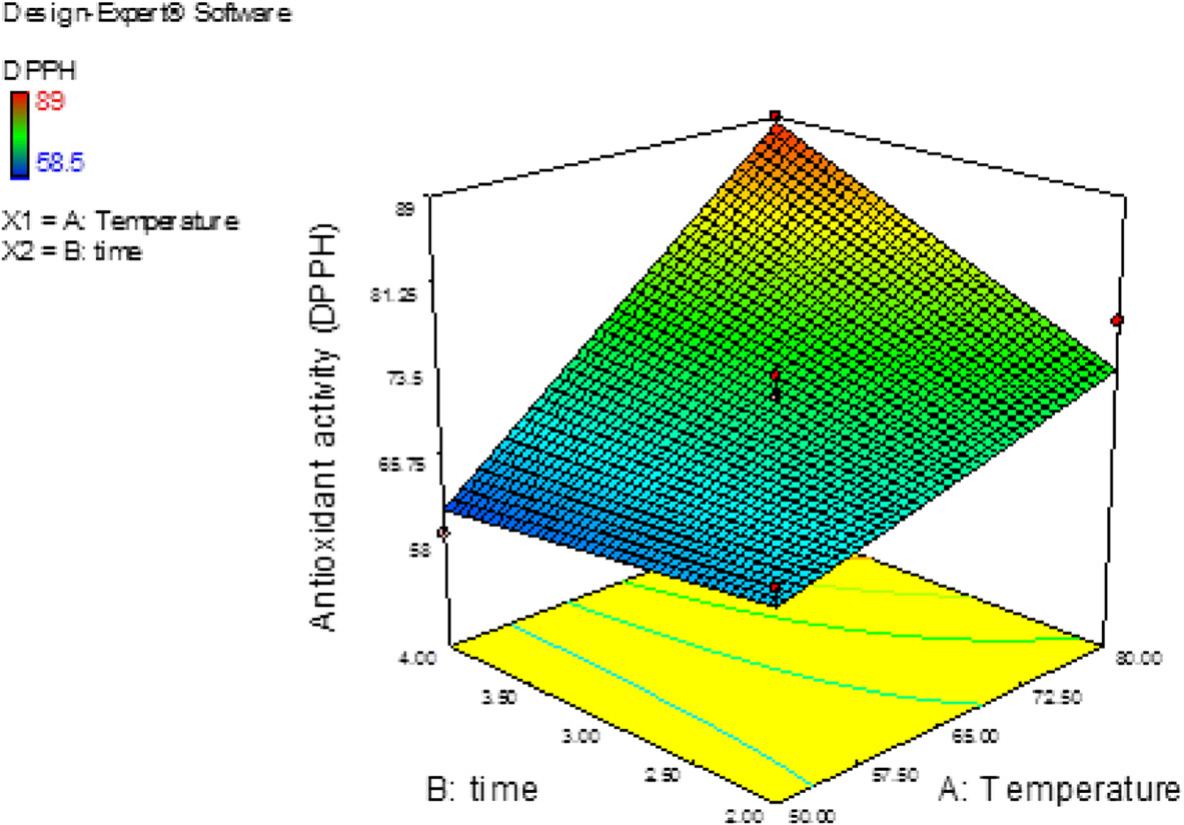
Response surface plot showing the effect of extraction temperature and time on antioxidant activity (DPPH) in *Z.officinale* var. *rubrum* Theilade rhizome extracts

The model F-value of 15.79 obtained for the antioxidant activity implies that the model is significant, and the “Lack of Fit *F* -value” of 1.24 implies that the Lack of Fit is not significant for the predicted model. Figure 3 shows the response surface plot for the relationship between the antioxidant activity (DPPH) and the extraction temperature and time. An increase in the DPPH activity was observed with increasing extraction temperature and time. The increase in the DPPH activity may be due to an increase in the 6-gingerol and 6-shogaol content in the extract.

### Correlation between 6-gingerol, 6-shogaol and DPPH activity

**Fig. 4:**
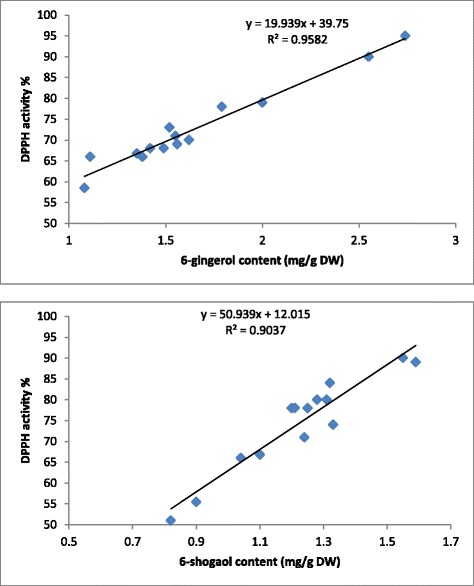
Linear regression line between DPPH activity, 6-gingerol and 6-shogaol content in *Z.officinale* var. *rubrum* Theilade rhizome extracts

Previous studies reported that the major bioactive constituents of ginger are gingerols and shogaols with high pharmacological activities [20, 21]. Herein, a significant (*P* < 0.01) correlation between the 6-gingerol and 6-shogaol content and antioxidant capacity of the ginger extract (R^2^ = 0.95 and 0.90) was observed (Fig. 4), which is in agreement with observations by Ali et al. [22], who reported that ginger extract with a high 6-gingerol and 6-shogaol content exhibited high free radical scavenging activity. Recent study by Pawar et al. [23] and Guo et al. [24] showed that there is an strong correlation between antioxidant activity of ginger and 6-gingerol content. Herein, 6-gingerol was also found to exhibit the most potent antioxidant properties, whereas 6-shogaol was the least potent. The results obtained in the current study provide additional evidence to support the assumption that gingerols are responsible for the antioxidant activity of ginger rhizome [25, 26]. The potent pharmaceutical quality of 6-gingerol may be attributed to its chemical structure. The predicted results were highly consistent with the experimental results obtained using the optimum extraction conditions predicted by the model, which validates the RSM model with good correlation.

### Optimization of response

In order to obtain ginger extract with a high content of 6-gingerol, 6-shogaol, and high antioxidant activity, the optimal reflux extraction conditions were determined based on the combination of both responses. Multiple graphical and numerical optimizations were carried out to determine the optimum level for the independent variables with desirable response goals. One optimal condition was obtained for all responses, which was reflux extraction at 76.9 °C for 3.4 h. Under the optimum conditions, the corresponding predicted response values for 6-gingerol, 6-shogaol, and the antioxidant activity were 2.89 mg/g DW, 1.85 mg/g DW, and 84.3 % respectively.

### Verification of the model

An experiment was performed for verify the adequacy of the developed extraction model, with the predicted optimum treatment conditions (76.9 °C and 3.4 h). Under this condition the obtained concentration for the 6-gingerol and 6-shogaol content, and the antioxidant activity were 2.92 and 1.88 mg/g DW, and 84.0 %, respectively (Table 4). The results of response surface analysis for the 6-gingerol and 6-shogaol content and antioxidant activity were verified by comparing the predicted values (2.89 mg/g DW, 1.85 mg/g DW, and 84.3 %) with the experimental values (2.92, 1.88 mg/g DW, and 84.0 %). The obtained results from verification experiment were in consent with the predicted values, because not significant (P > 0.05) difference was observed between the verification experimental and the predicted values.

**Table 4:** Verification of model for predicted optimum treatment conditions (78.9 °C and 3.8 h)

	6-gingerol (mg/g DW)	6-shogaol (mg/g DW)	Antioxidant activity (%)
Predicted	2.89	1.85	84.3
Experimental	2.92	1.88	84.0

### Evaluation of anticancer activity of optimized and unoptimized ginger extract

**Fig. 5:**
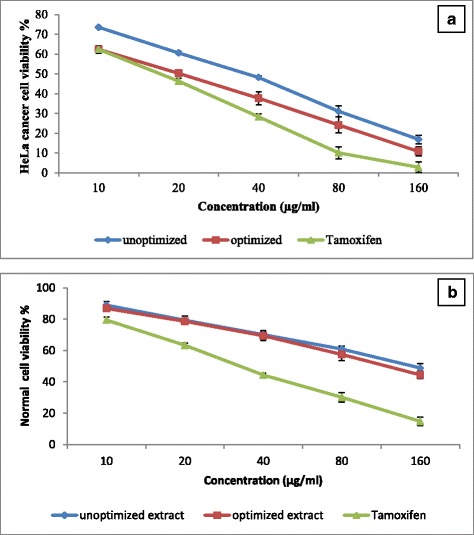
Dose-dependent anticancer activity of *Z.officinale* var. *rubrum* Theilade rhizome extracts against HeLa cell line (**a**) and normal cell viability (**b**). Tamoxifen was used as a positive control. Bars represent standard error of means

Optimized and unoptimized extracts of *Z.officinale* var. *rubrum* Theilade rhizome were used in order to evaluate the anticancer activity against HeLa cancer cell lines. Preliminary screening showed that rhizomes extracts exhibited a significant anticancer activity against HeLa cancer cells at concentration of 40$\mu$g/mL with the inhibition rate of 51.8 and 62.3 % from unoptimized and optimized extracts, respectively (Fig. 5a). HeLa cells showed 71.7 % inhibition when treated with tamoxifen (positive control) at the concentration of 40$\mu$g/mL. Furthermore, with optimizing of reflux extraction anticancer activity of extracts were enhanced about 16.8 %. The half maximal inhibitory concentration (IC_50_) value of optimized and unoptimized extract was found at concentration of 20.9 and 38.4 μg/mL respectively. The IC_50_ value for tamoxifen was observed at concentration of 16.4 μg/mL. As shown in Fig. 5b the normal cells treated with the optimized and unoptimized extract of *Z.officinale* var. *rubrum* Theilade rhizome showed 70.13 and 69.43 % of viability at concentration of 40$\mu$g/mL, respectively. According to the obtained results, optimized and unoptimized extracts showed non toxic effects at the concentrations bellow 120 $\mu$g/mL. 6-gingerol and 6-shogale were reported as a potent anticancer compound in ginger [27–30]. The results of previous studies showed that 6-shogaol is able to kill fifty percent of Hela cancer cell line at concentration of 14.75 μM [31]. Then, it could be conducted that enhancement of anticancer activity of optimized extract could be related to rising of 6-gingerol and 6-shogaol content in extract.

## Conclusion

Response surface methodology was successfully implemented for the optimization of the experimental conditions for achieving high 6-gingerol and 6-shogaol content and antioxidant activity in ginger extracts. The results indicate that the extraction temperature and time affected the extraction yields of 6-gingerol and 6-shogaol significantly, and consequently the antioxidant activity of the extracts. Reflux extraction at 76.9 °C for 3.4 h was determined to be the most efficient condition for the extraction of 6-gingerol and 6-shogaol from *Z.officinale* var. *rubrum* Theilade rhizome to provide high antioxidant activity. Optimized extract showed a more distinct scavenging activity against the DPPH and also showed significant anticancer activities toward HeLa cancer cell lines in a concentration of 40 μg/mL without toxicity to normal cells.
